# 2-Isopropyl-6-methyl-4-oxo-3,4-dihydro­pyrimidin-1-ium 2-carb­oxy-4,6-dinitro­phenolate monohydrate

**DOI:** 10.1107/S1600536810042571

**Published:** 2010-10-30

**Authors:** Madhukar Hemamalini, Hoong-Kun Fun

**Affiliations:** a X-ray Crystallography Unit, School of Physics, Universiti Sains Malaysia, 11800 USM, Penang, Malaysia

## Abstract

In the title mol­ecular salt, C_8_H_13_N_2_O^+^·C_7_H_3_N_2_O_7_^−^·H_2_O, the pyrimidinium cation is essentially planar, with a maximum deviation of 0.009 (1) Å. The cation undergoes an enol–keto tautomerism during the crystallization. In the crystal, the ion pairs and water mol­ecules are connected *via* O—H⋯O, N—H⋯O and C—H⋯O hydrogen bonds, forming two-dimensional networks parallel to the *bc* plane. There is an intra­molecular O—H⋯O hydrogen bond in the 3,5-dinitro­salicylate anion, which generates an *S*(6) ring motif.

## Related literature

For applications of pyrimidine derivaties, see: Condon *et al.* (1993 ▶); Maeno *et al.* (1990 ▶); Gilchrist (1997 ▶). For a related structure, see: Hemamalini & Fun (2010 ▶). For hydrogen-bond motifs, see: Bernstein *et al.* (1995 ▶). For bond-length data, see: Allen *et al.* (1987 ▶). For the stability of the temperature controller used in the data collection, see: Cosier & Glazer (1986 ▶).
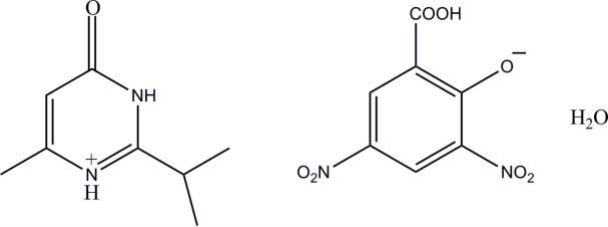


## Experimental

### 

#### Crystal data

C_8_H_13_N_2_O^+^·C_7_H_3_N_2_O_7_^−^·H_2_O
*M* *_r_* = 398.33
Triclinic, 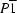

*a* = 6.6691 (3) Å
*b* = 11.3831 (4) Å
*c* = 12.2900 (5) Å
α = 89.727 (2)°
β = 76.771 (2)°
γ = 76.930 (2)°
*V* = 883.62 (6) Å^3^
*Z* = 2
Mo *K*α radiation
μ = 0.13 mm^−1^
*T* = 100 K
0.52 × 0.13 × 0.10 mm

#### Data collection

Bruker SMART APEXII CCD area-detector diffractometer
Absorption correction: multi-scan (*SADABS*; Bruker, 2009 ▶) *T*_min_ = 0.937, *T*_max_ = 0.987
17014 measured reflections
4061 independent reflections
3279 reflections with *I* > 2σ(*I*)
*R*_int_ = 0.030

#### Refinement

*R*[*F* ^2^ > 2σ(*F* ^2^)] = 0.040
*wR*(*F* ^2^) = 0.105
*S* = 1.03
4061 reflections
273 parameters
H atoms treated by a mixture of independent and constrained refinement
Δρ_max_ = 0.53 e Å^−3^
Δρ_min_ = −0.30 e Å^−3^

### 

Data collection: *APEX2* (Bruker, 2009 ▶); cell refinement: *SAINT* (Bruker, 2009 ▶); data reduction: *SAINT*; program(s) used to solve structure: *SHELXTL* (Sheldrick, 2008 ▶); program(s) used to refine structure: *SHELXTL*; molecular graphics: *SHELXTL*; software used to prepare material for publication: *SHELXTL* and *PLATON* (Spek, 2009 ▶).

## Supplementary Material

Crystal structure: contains datablocks global, I. DOI: 10.1107/S1600536810042571/fj2355sup1.cif

Structure factors: contains datablocks I. DOI: 10.1107/S1600536810042571/fj2355Isup2.hkl

Additional supplementary materials:  crystallographic information; 3D view; checkCIF report

## Figures and Tables

**Table 1:** Hydrogen-bond geometry (Å, °)

*D*—H⋯*A*	*D*—H	H⋯*A*	*D*⋯*A*	*D*—H⋯*A*
N3—H1*N*3⋯O6^i^	0.91 (2)	1.817 (19)	2.7180 (16)	170 (2)
N4—H1*N*4⋯O1*W*	0.90 (2)	1.84 (2)	2.7309 (17)	171 (2)
O1*W*—H2*W*1⋯O1^ii^	0.85 (2)	1.97 (2)	2.7878 (16)	162 (2)
O1*W*—H1*W*1⋯O3^iii^	0.84 (2)	2.11 (2)	2.9381 (17)	170 (2)
O7—H7⋯O1	0.82	1.67	2.4370 (16)	156
C9—H9*A*⋯O5^iv^	0.93	2.54	3.4312 (18)	161
C12—H12*A*⋯O7^i^	0.98	2.41	3.3023 (18)	152
C14—H14*B*⋯O4^v^	0.96	2.60	3.2318 (19)	124
C15—H15*C*⋯O3^vi^	0.96	2.60	3.471 (2)	152

Symmetry codes: (i) 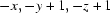; (ii) 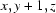; (iii) 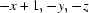; (iv) 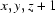; (v) 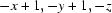; (vi) 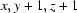.

**Fig. 1.**
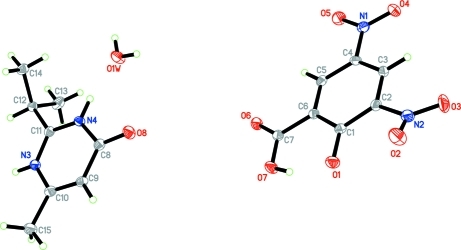
The asymmetric unit of the title compound. Displacement ellipsoids are drawn at the 50% probability level.

**Fig. 2.**
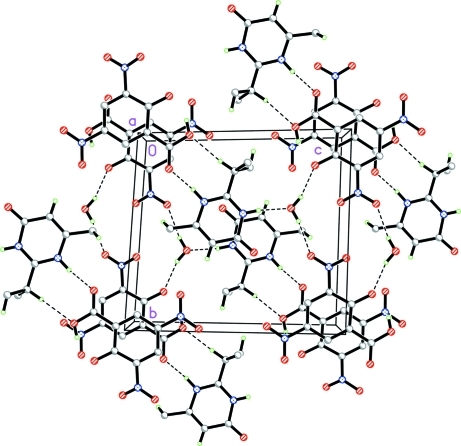
The crystal packing of the title compound, showing hydrogen-bonded (dashed lines) networks. H atoms not involved in the interactions have been omitted for clarity.

## supplementary crystallographic information

### Comment

Pyrimidine derivatives are very important molecules in biology and have many application in the areas of pesticide and pharmaceutical agents (Condon *et al.*, 1993). For example, imazosulfuron, ethirmol and mepanipyrim have been commercialized as agrochemicals (Maeno *et al.*, 1990).  Pyrimidine derivatives have also been developed as antiviral agents, such as AZT, which is the most widely-used anti-AIDS drug (Gilchrist, 1997). The nitro-substituted aromatic acid 3,5-dinitrosalicylic acid (DNSA) has proven potential for formation of proton-transfer compounds, particularly because of its acid strength (pK_a_ = 2.18), its interactive ortho-related phenolic substituent group together with the nitro substituents which have potential for both π···π interactions as well as hydrogen-bonding interactions.  Since our aim is to study some interesting hydrogen bonding interactions, the crystal structure of the title compound is presented here.

The asymmetric unit of (I) (Fig 1), contains a 2-isopropyl-6-methyl pyrimidinium-4(3*H*)-one cation, a 3,5-dinitrosalicylate anion and a water molecule.  In the  cation, the proton transfer from the hydroxyl group of the anion to the N4 atom leads to a slight increase in the C8—N4—C11 angle to 124.54 (12)°, compared to 116.83 (8)° in (Hemamalini & Fun, 2010).  The phenol oxygen atoms are bent slightly away from the mean plane of the benzene ring [torsion angle O1-C7-C8-C9 = 177.02 (13)°]. The Pyrimidine ring is essentially planar, with a maximum deviation of 0.009 (1) Å for atom N4.  The bond lengths (Allen *et al.*, 1987) and angles are normal. The cation undergoes an enol-keto tautomerism during the crystallization. Similar tautomerism was also observed in the crystal structure of 2-Isopropyl-6-methylpyrimidinium-4(3*H*)-one (Hemamalini & Fun, 2010).

In the crystals structure (Fig. 2), the ion pairs and water molecules are connected via N3—H1N3···O6, N4—H1N4···O1W, O1W—H2W1···O1, O1W—H1W1···O3, C9—H9A···O5, C12—H12A···O7, C14—H14B···O4 and C15—H15C···O3 hydrogen bonds, forming two-dimensional networks parallel to the *bc* plane.  There is an intramolecular O7—H7···O1 hydrogen bond in the 3,5-dinitrosalicylate anion which generates an *S*(6) (Bernstein *et al.*, 1995) ring motif.

### Experimental

A hot methanol solution (20 ml) of 2-isopropyl-4-hydroxy-6-methylpyrimidine (46 mg, Aldrich) and 3,5-dinitrosalicylic acid (58 mg, Merck) were mixed and warmed over a heating magnetic stirrer for a few minutes. The resulting solution was allowed to cool slowly at room temperature and yellow blocks of (I) appeared after a few days.

### Refinement

Atoms H1N3, H1N4, H2W1 and H1W1 were located from a difference Fourier map and were refined freely [N–H = 0.90 (2)– 0.91 (2) Å and O–H = 0.82–0.85 (2) Å]. The remaining hydrogen atoms were positioned geometrically [C–H = 0.93 or 0.96 Å] and were refined using a riding model, with *U*_iso_(H) = 1.2 or 1.5 *U*_eq_(C,O). A rotating group model was used for the methyl group.

### Crystal data

**Table:**

C_8_H_13_N_2_O^+^·C_7_H_3_N_2_O_7_^−^·H_2_O	*Z* = 2
*M**_r_* = 398.33	*F*(000) = 416
Triclinic, *P*1	*D*_x_ = 1.497 Mg m^−^^3^
Hall symbol: -P 1	Mo *K*α radiation, λ = 0.71073 Å
*a* = 6.6691 (3) Å	Cell parameters from 6994 reflections
*b* = 11.3831 (4) Å	θ = 2.4–31.6°
*c* = 12.2900 (5) Å	µ = 0.13 mm^−^^1^
α = 89.727 (2)°	*T* = 100 K
β = 76.771 (2)°	Block, yellow
γ = 76.930 (2)°	0.52 × 0.13 × 0.10 mm
*V* = 883.62 (6) Å^3^	

### Data collection

**Table:**

Bruker SMART APEXII CCD area-detector diffractometer	4061 independent reflections
Radiation source: fine-focus sealed tube	3279 reflections with *I* > 2σ(*I*)
graphite	*R*_int_ = 0.030
φ and ω scans	θ_max_ = 27.5°, θ_min_ = 1.7°
Absorption correction: multi-scan (*SADABS*; Bruker, 2009)	*h* = −8→8
*T*_min_ = 0.937, *T*_max_ = 0.987	*k* = −14→12
17014 measured reflections	*l* = −15→15

### Refinement

**Table:**

Refinement on *F*^2^	Primary atom site location: structure-invariant direct methods
Least-squares matrix: full	Secondary atom site location: difference Fourier map
*R*[*F*^2^ > 2σ(*F*^2^)] = 0.040	Hydrogen site location: inferred from neighbouring sites
*wR*(*F*^2^) = 0.105	H atoms treated by a mixture of independent and constrained refinement
*S* = 1.03	*w* = 1/[σ^2^(*F*_o_^2^) + (0.0477*P*)^2^ + 0.3946*P*] where *P* = (*F*_o_^2^ + 2*F*_c_^2^)/3
4061 reflections	(Δ/σ)_max_ < 0.001
273 parameters	Δρ_max_ = 0.53 e Å^−^^3^
0 restraints	Δρ_min_ = −0.30 e Å^−^^3^

### Special details

**Table:**

Experimental. The crystal was placed in the cold stream of an Oxford Cryosystems Cobra open-flow nitrogen cryostat (Cosier & Glazer, 1986) operating at 100.0 (1) K.
Geometry. All s.u.'s (except the s.u. in the dihedral angle between two l.s. planes) are estimated using the full covariance matrix. The cell s.u.'s are taken into account individually in the estimation of s.u.'s in distances, angles and torsion angles; correlations between s.u.'s in cell parameters are only used when they are defined by crystal symmetry. An approximate (isotropic) treatment of cell s.u.'s is used for estimating s.u.'s involving l.s. planes.
Refinement. Refinement of F^2^ against ALL reflections. The weighted R-factor wR and goodness of fit S are based on F^2^, conventional R-factors R are based on F, with F set to zero for negative F^2^. The threshold expression of F^2^ > 2σ(F^2^) is used only for calculating R-factors(gt) etc. and is not relevant to the choice of reflections for refinement. R-factors based on F^2^ are statistically about twice as large as those based on F, and R- factors based on ALL data will be even larger.

### Fractional atomic coordinates and isotropic or equivalent isotropic displacement parameters (Å^2^)

**Table:**

	*x*	*y*	*z*	*U*_iso_*/*U*_eq_	
O1	0.30537 (18)	−0.17099 (10)	0.14974 (9)	0.0223 (2)	
O2	0.5667 (2)	−0.35714 (10)	0.01344 (10)	0.0310 (3)	
O3	0.4865 (2)	−0.36692 (10)	−0.14689 (10)	0.0308 (3)	
O4	0.36257 (17)	−0.00307 (10)	−0.33888 (9)	0.0231 (3)	
O5	0.23770 (18)	0.16989 (10)	−0.24573 (9)	0.0251 (3)	
O6	0.07237 (17)	0.19149 (9)	0.16326 (9)	0.0200 (2)	
O7	0.14505 (18)	0.02269 (10)	0.25242 (9)	0.0213 (2)	
H7	0.1960	−0.0493	0.2360	0.032*	
O8	0.31892 (18)	0.38518 (9)	0.41955 (9)	0.0220 (2)	
N1	0.30166 (19)	0.05932 (11)	−0.25097 (10)	0.0182 (3)	
N2	0.4862 (2)	−0.31082 (11)	−0.06121 (11)	0.0206 (3)	
N3	0.11901 (19)	0.64262 (11)	0.66450 (10)	0.0148 (3)	
N4	0.23843 (18)	0.58272 (10)	0.47925 (10)	0.0144 (3)	
C1	0.3103 (2)	−0.11927 (13)	0.05577 (12)	0.0151 (3)	
C2	0.3897 (2)	−0.18173 (13)	−0.05146 (12)	0.0156 (3)	
C3	0.3840 (2)	−0.12442 (13)	−0.15046 (12)	0.0158 (3)	
H3A	0.4330	−0.1682	−0.2188	0.019*	
C4	0.3044 (2)	−0.00117 (13)	−0.14625 (12)	0.0153 (3)	
C5	0.2283 (2)	0.06623 (13)	−0.04535 (12)	0.0150 (3)	
H5A	0.1774	0.1494	−0.0446	0.018*	
C6	0.2289 (2)	0.00879 (13)	0.05385 (12)	0.0139 (3)	
C7	0.1427 (2)	0.08136 (13)	0.16144 (12)	0.0157 (3)	
C8	0.2598 (2)	0.45827 (13)	0.49893 (12)	0.0158 (3)	
C9	0.2071 (2)	0.43350 (13)	0.61585 (12)	0.0159 (3)	
H9A	0.2214	0.3538	0.6364	0.019*	
C10	0.1374 (2)	0.52333 (13)	0.69601 (12)	0.0159 (3)	
C11	0.1694 (2)	0.67070 (13)	0.55878 (12)	0.0142 (3)	
C12	0.1447 (2)	0.80024 (12)	0.52940 (12)	0.0151 (3)	
H12A	0.1079	0.8501	0.5990	0.018*	
C13	−0.0376 (2)	0.83580 (14)	0.47027 (14)	0.0221 (3)	
H13A	−0.1654	0.8227	0.5181	0.033*	
H13B	−0.0048	0.7874	0.4020	0.033*	
H13C	−0.0568	0.9195	0.4537	0.033*	
C14	0.3510 (2)	0.82287 (13)	0.45724 (13)	0.0192 (3)	
H14A	0.4628	0.7980	0.4955	0.029*	
H14B	0.3331	0.9073	0.4438	0.029*	
H14C	0.3864	0.7774	0.3871	0.029*	
C15	0.0772 (3)	0.50606 (14)	0.81836 (13)	0.0215 (3)	
H15A	0.0965	0.4214	0.8310	0.032*	
H15B	−0.0686	0.5459	0.8474	0.032*	
H15C	0.1647	0.5398	0.8555	0.032*	
O1W	0.3284 (2)	0.60970 (11)	0.25362 (10)	0.0271 (3)	
H1N3	0.066 (3)	0.7030 (18)	0.7187 (16)	0.028 (5)*	
H1N4	0.271 (3)	0.5992 (18)	0.4063 (17)	0.032 (5)*	
H2W1	0.339 (3)	0.667 (2)	0.2096 (17)	0.035 (5)*	
H1W1	0.377 (4)	0.544 (2)	0.2154 (19)	0.044 (6)*	

### Atomic displacement parameters (Å^2^)

**Table:**

	*U*^11^	*U*^22^	*U*^33^	*U*^12^	*U*^13^	*U*^23^
O1	0.0296 (6)	0.0173 (5)	0.0173 (5)	−0.0039 (5)	−0.0015 (4)	0.0032 (4)
O2	0.0424 (7)	0.0207 (6)	0.0229 (6)	0.0050 (5)	−0.0056 (5)	0.0036 (5)
O3	0.0487 (8)	0.0171 (6)	0.0231 (6)	−0.0034 (5)	−0.0054 (5)	−0.0076 (5)
O4	0.0289 (6)	0.0268 (6)	0.0137 (5)	−0.0082 (5)	−0.0031 (4)	−0.0002 (5)
O5	0.0344 (6)	0.0173 (6)	0.0230 (6)	−0.0043 (5)	−0.0075 (5)	0.0060 (5)
O6	0.0244 (6)	0.0138 (5)	0.0183 (5)	−0.0014 (4)	−0.0006 (4)	−0.0027 (4)
O7	0.0287 (6)	0.0153 (5)	0.0149 (5)	−0.0002 (5)	0.0004 (4)	−0.0004 (4)
O8	0.0323 (6)	0.0137 (5)	0.0175 (6)	−0.0029 (5)	−0.0031 (5)	−0.0019 (4)
N1	0.0184 (6)	0.0198 (7)	0.0179 (7)	−0.0074 (5)	−0.0047 (5)	0.0035 (5)
N2	0.0240 (7)	0.0153 (6)	0.0186 (7)	−0.0035 (5)	0.0018 (5)	0.0001 (5)
N3	0.0166 (6)	0.0122 (6)	0.0140 (6)	−0.0024 (5)	−0.0017 (5)	−0.0006 (5)
N4	0.0171 (6)	0.0116 (6)	0.0130 (6)	−0.0026 (5)	−0.0013 (5)	0.0010 (5)
C1	0.0136 (7)	0.0169 (7)	0.0151 (7)	−0.0059 (5)	−0.0014 (5)	0.0016 (6)
C2	0.0161 (7)	0.0122 (7)	0.0174 (7)	−0.0032 (5)	−0.0020 (5)	−0.0006 (5)
C3	0.0166 (7)	0.0179 (7)	0.0137 (7)	−0.0072 (6)	−0.0016 (5)	−0.0019 (6)
C4	0.0152 (7)	0.0195 (7)	0.0130 (7)	−0.0076 (6)	−0.0032 (5)	0.0034 (6)
C5	0.0132 (7)	0.0135 (7)	0.0186 (7)	−0.0046 (5)	−0.0028 (5)	0.0008 (6)
C6	0.0116 (6)	0.0149 (7)	0.0151 (7)	−0.0043 (5)	−0.0014 (5)	−0.0007 (5)
C7	0.0134 (7)	0.0169 (7)	0.0159 (7)	−0.0047 (5)	−0.0003 (5)	−0.0009 (6)
C8	0.0156 (7)	0.0132 (7)	0.0182 (7)	−0.0028 (5)	−0.0035 (5)	0.0004 (6)
C9	0.0178 (7)	0.0112 (7)	0.0181 (7)	−0.0030 (5)	−0.0036 (6)	0.0036 (6)
C10	0.0147 (7)	0.0157 (7)	0.0175 (7)	−0.0039 (6)	−0.0041 (5)	0.0033 (6)
C11	0.0117 (6)	0.0143 (7)	0.0163 (7)	−0.0028 (5)	−0.0027 (5)	−0.0001 (5)
C12	0.0183 (7)	0.0105 (7)	0.0154 (7)	−0.0033 (5)	−0.0019 (5)	−0.0001 (5)
C13	0.0222 (8)	0.0136 (7)	0.0319 (9)	−0.0033 (6)	−0.0099 (7)	0.0055 (6)
C14	0.0203 (7)	0.0138 (7)	0.0221 (8)	−0.0051 (6)	−0.0008 (6)	0.0007 (6)
C15	0.0267 (8)	0.0189 (8)	0.0176 (8)	−0.0048 (6)	−0.0031 (6)	0.0021 (6)
O1W	0.0475 (8)	0.0128 (6)	0.0158 (6)	−0.0038 (5)	−0.0005 (5)	0.0009 (5)

### Geometric parameters (Å, °)

**Table:**

O1—C1	1.2909 (17)	C5—C6	1.3811 (19)
O2—N2	1.2250 (17)	C5—H5A	0.9300
O3—N2	1.2334 (17)	C6—C7	1.486 (2)
O4—N1	1.2299 (16)	C8—C9	1.441 (2)
O5—N1	1.2311 (16)	C9—C10	1.348 (2)
O6—C7	1.2335 (17)	C9—H9A	0.9300
O7—C7	1.3010 (17)	C10—C15	1.489 (2)
O7—H7	0.8200	C11—C12	1.4981 (19)
O8—C8	1.2177 (18)	C12—C14	1.5314 (19)
N1—C4	1.4586 (18)	C12—C13	1.533 (2)
N2—C2	1.4581 (18)	C12—H12A	0.9800
N3—C11	1.3221 (18)	C13—H13A	0.9600
N3—C10	1.3965 (18)	C13—H13B	0.9600
N3—H1N3	0.91 (2)	C13—H13C	0.9600
N4—C11	1.3285 (19)	C14—H14A	0.9600
N4—C8	1.4166 (18)	C14—H14B	0.9600
N4—H1N4	0.90 (2)	C14—H14C	0.9600
C1—C2	1.429 (2)	C15—H15A	0.9600
C1—C6	1.438 (2)	C15—H15B	0.9600
C2—C3	1.382 (2)	C15—H15C	0.9600
C3—C4	1.380 (2)	O1W—H2W1	0.85 (2)
C3—H3A	0.9300	O1W—H1W1	0.84 (2)
C4—C5	1.389 (2)		
			
C7—O7—H7	109.5	N4—C8—C9	113.61 (12)
O4—N1—O5	124.05 (12)	C10—C9—C8	121.41 (13)
O4—N1—C4	118.14 (12)	C10—C9—H9A	119.3
O5—N1—C4	117.81 (12)	C8—C9—H9A	119.3
O2—N2—O3	123.54 (13)	C9—C10—N3	118.94 (13)
O2—N2—C2	119.12 (12)	C9—C10—C15	124.98 (13)
O3—N2—C2	117.31 (12)	N3—C10—C15	116.08 (13)
C11—N3—C10	122.35 (13)	N3—C11—N4	119.11 (13)
C11—N3—H1N3	119.1 (12)	N3—C11—C12	120.22 (13)
C10—N3—H1N3	118.5 (12)	N4—C11—C12	120.66 (12)
C11—N4—C8	124.54 (12)	C11—C12—C14	111.37 (12)
C11—N4—H1N4	121.1 (12)	C11—C12—C13	109.34 (11)
C8—N4—H1N4	114.3 (12)	C14—C12—C13	111.33 (12)
O1—C1—C2	124.18 (13)	C11—C12—H12A	108.2
O1—C1—C6	120.48 (13)	C14—C12—H12A	108.2
C2—C1—C6	115.33 (12)	C13—C12—H12A	108.2
C3—C2—C1	122.73 (13)	C12—C13—H13A	109.5
C3—C2—N2	116.52 (13)	C12—C13—H13B	109.5
C1—C2—N2	120.74 (12)	H13A—C13—H13B	109.5
C4—C3—C2	118.90 (13)	C12—C13—H13C	109.5
C4—C3—H3A	120.5	H13A—C13—H13C	109.5
C2—C3—H3A	120.5	H13B—C13—H13C	109.5
C3—C4—C5	121.76 (13)	C12—C14—H14A	109.5
C3—C4—N1	118.76 (13)	C12—C14—H14B	109.5
C5—C4—N1	119.48 (13)	H14A—C14—H14B	109.5
C6—C5—C4	119.48 (13)	C12—C14—H14C	109.5
C6—C5—H5A	120.3	H14A—C14—H14C	109.5
C4—C5—H5A	120.3	H14B—C14—H14C	109.5
C5—C6—C1	121.77 (13)	C10—C15—H15A	109.5
C5—C6—C7	119.05 (13)	C10—C15—H15B	109.5
C1—C6—C7	119.18 (12)	H15A—C15—H15B	109.5
O6—C7—O7	122.30 (13)	C10—C15—H15C	109.5
O6—C7—C6	121.12 (13)	H15A—C15—H15C	109.5
O7—C7—C6	116.58 (12)	H15B—C15—H15C	109.5
O8—C8—N4	119.20 (13)	H2W1—O1W—H1W1	108 (2)
O8—C8—C9	127.19 (13)		
			
O1—C1—C2—C3	177.02 (13)	O1—C1—C6—C7	1.1 (2)
C6—C1—C2—C3	−1.7 (2)	C2—C1—C6—C7	179.94 (12)
O1—C1—C2—N2	−4.3 (2)	C5—C6—C7—O6	−0.5 (2)
C6—C1—C2—N2	176.93 (12)	C1—C6—C7—O6	179.66 (13)
O2—N2—C2—C3	153.14 (14)	C5—C6—C7—O7	179.34 (12)
O3—N2—C2—C3	−24.99 (19)	C1—C6—C7—O7	−0.53 (19)
O2—N2—C2—C1	−25.6 (2)	C11—N4—C8—O8	177.87 (13)
O3—N2—C2—C1	156.26 (13)	C11—N4—C8—C9	−2.37 (19)
C1—C2—C3—C4	2.0 (2)	O8—C8—C9—C10	−178.07 (15)
N2—C2—C3—C4	−176.69 (12)	N4—C8—C9—C10	2.18 (19)
C2—C3—C4—C5	−0.6 (2)	C8—C9—C10—N3	−0.8 (2)
C2—C3—C4—N1	179.06 (12)	C8—C9—C10—C15	179.28 (13)
O4—N1—C4—C3	2.98 (19)	C11—N3—C10—C9	−0.6 (2)
O5—N1—C4—C3	−177.24 (13)	C11—N3—C10—C15	179.26 (13)
O4—N1—C4—C5	−177.33 (12)	C10—N3—C11—N4	0.5 (2)
O5—N1—C4—C5	2.45 (19)	C10—N3—C11—C12	179.41 (12)
C3—C4—C5—C6	−1.0 (2)	C8—N4—C11—N3	1.1 (2)
N1—C4—C5—C6	179.34 (12)	C8—N4—C11—C12	−177.78 (12)
C4—C5—C6—C1	1.2 (2)	N3—C11—C12—C14	126.27 (14)
C4—C5—C6—C7	−178.63 (12)	N4—C11—C12—C14	−54.87 (17)
O1—C1—C6—C5	−178.73 (13)	N3—C11—C12—C13	−110.27 (15)
C2—C1—C6—C5	0.07 (19)	N4—C11—C12—C13	68.59 (17)

### Hydrogen-bond geometry (Å, °)

**Table:**

*D*—H···*A*	*D*—H	H···*A*	*D*···*A*	*D*—H···*A*
N3—H1N3···O6^i^	0.91 (2)	1.817 (19)	2.7180 (16)	170 (2)
N4—H1N4···O1W	0.90 (2)	1.84 (2)	2.7309 (17)	171 (2)
O1W—H2W1···O1^ii^	0.85 (2)	1.97 (2)	2.7878 (16)	162 (2)
O1W—H1W1···O3^iii^	0.84 (2)	2.11 (2)	2.9381 (17)	170 (2)
O7—H7···O1	0.82	1.67	2.4370 (16)	156
C9—H9A···O5^iv^	0.93	2.54	3.4312 (18)	161
C12—H12A···O7^i^	0.98	2.41	3.3023 (18)	152
C14—H14B···O4^v^	0.96	2.60	3.2318 (19)	124
C15—H15C···O3^vi^	0.96	2.60	3.471 (2)	152

Symmetry codes: (i) −*x*, −*y*+1, −*z*+1; (ii) *x*, *y*+1, *z*; (iii) −*x*+1, −*y*, −*z*; (iv) *x*, *y*, *z*+1; (v) −*x*+1, −*y*+1, −*z*; (vi) *x*, *y*+1, *z*+1.
